# Small Dielectric Spheres with High Refractive Index as New Multifunctional Elements for Optical Devices

**DOI:** 10.1038/srep12288

**Published:** 2015-07-23

**Authors:** Michael I. Tribelsky, Jean-Michel Geffrin, Amelie Litman, Christelle Eyraud, Fernando Moreno

**Affiliations:** 1 Lomonosov Moscow State University, Russia; 2 Moscow State University of Information Technologies, Radioengineering and Electronics MIREA, Moscow, Russia; 3 Aix-Marseille Université, CNRS, Centrale Marseille, Institut Fresnel UMR 7249, 13013 Marseille, France; 4 Group of Optics. Department of Applied Physics. University of Cantabria, Spain

## Abstract

The future of ultra-fast optical communication systems is inevitably connected with progress in optical circuits and nanoantennas. One of the key points of this progress is the creation of elementary components of optical devices with scattering diagrams tailored for redirecting the incident light in a desired manner. Here we demonstrate theoretically and experimentally that a small, simple, spatially homogeneous dielectric subwavelength sphere with a high refractive index and low losses (as some semiconductors in the visible or near infrared region) exhibits properties allowing to utilize it as a new multifunctional element for the mentioned devices. This can be achieved by taking advantage of the coherent effects between dipolar and multipolar modes, which produce *anomalous* scattering effects. The effects open a new way to control the *directionality* of the scattered light. The *directional* tuning can be obtained in a practical way just by a change in the frequency of the incident wave, and/or by a well-chosen diameter of the sphere. Dielectric nanoparticles with the required optical properties in the VIS-NIR may be now readily fabricated. These particles could be an efficient alternative to the widely discussed scattering units with a more complicated design.

The recent extensive research of light scattering by various plasmonic nanostructures^1^,^2^,^3^,^4^,^5^,^6^,^7^,^8^,^9^,^10^,^11^,^12^ is explained by the hope of numerous future applications of these structures, especially in ultra-fast optical communication systems and nanoantennas. The final goal of this research is to create physical grounds for the design and fabrication of optical scattering elements capable to control the intensity, polarization, phase and directionality of the scattered radiation in rather wide limits. In principle, this can be achieved by selective excitation of different plasmonic eigenmodes and their controlled interference.

Especially promising in this sense has been the *anomalous scattering* introduced recently in a series of publications of one of the authors^13^,^14^,^15^. The phenomenon may be observed in light scattering by small (relative to the incident light wavelength) plasmonic particles with weak dissipation. Despite the smallness of the particles, the anomalous scattering has nothing in common with the conventional Rayleigh scattering, being in seeming violent contradiction with what is written in any textbook of optics, see, for instance, Ref. 16. Specifically, it is characterized by the *inverted hierarchy of resonances*, when the quadrupole plasmon resonance is more pronounced than the dipole, octupole is more pronounced than the quadrupole, etc.^13^. Another of its peculiarity is a very complicated circulation of energy in the near field zone, which brings about optical vortices and other singularities in the Poynting vector field^14^. These features of the *anomalous scattering* provide solid grounds to manipulate the scattered radiation and to tailor desired scattering diagrams.

However, up to now, the *anomalous scattering* does not have the convincing experimental evidence. The main obstacle in the way to get it is the fact that the phenomenon comes into play when the dissipative losses are so small that the radiative damping becomes dominant^17^. Unfortunately, most metals have large dissipation at the frequencies of the plasmon resonances. The mentioned necessary condition for the anomalous scattering to arise holds for aluminum, potassium and sodium^13^,^14^. Aluminum easily gets oxidized and shows interband transitions in the NIR. The other metals are chemically very aggressive, so it is difficult to envisage practical applications of particles made of these metals.

On the other hand, dielectric materials often may have very weak dissipation in a wide spectral range. Dielectrics do not exhibit plasmon resonances, since in contrast to metal they do not have free electron plasma, but they exhibit other Mie’s resonances, which do not require a finite electric conductivity. Then, a natural question arises: “May an analog of the *anomalous scattering* be observed in light scattering by a dielectric particle?”

The answer is affirmative and the observation of such a scattering by a dielectric sphere is reported in the present paper. We will show that a small, simple, spatially homogeneous dielectric subwavelength sphere with a high refractive index and low losses exhibits properties allowing to utilize it as a new multifunctional element of various optical devices. The switch from one function to another may be achieved just by a change in the frequency of the incident wave, and/or by a well-chosen diameter of the sphere. In the VIS-NIR range of the spectrum, such particles could be made, for instance, of silicon^18^, germanium^19^, or other semiconductor compounds^20^ by means of different micro- and/or nanolitography techniques, or “molded” to spherical shape on a substrate by either chemical vapor deposition^21^ or laser-induced transfer techniques^22^. These particles could be an efficient alternative to the widely discussed scattering systems with a more complicated design^8^,^9^,^10^. Here, we demonstrate theoretically and experimentally the possibility of selective excitation and controlled interference of dipolar and quadrupolar modes (both electric and magnetic) by irradiating the subwavelength particles with a plane electromagnetic wave. It allows us to tailor unusual, essentially anisotropic scattering diagrams almost “à la carte”. Our results give new insights into the fundamental problem of light scattering and provide research perspectives towards revolutionary new technologies in subwavelength optics.

Unfortunately the study of light scattering by an individual nanoparticle is a challenging problem due to its small scale and low intensity of the light it scatters^6^. In fact, in the visible part of the spectrum, researches are focused in ensembles of particles and in general these studies and conclusions are centered in the analysis of experimentally measured scattering and extinction cross sections^24^. In the nanometric range of the particle sizes and for VIS-NIR light frequencies, these parameters can be measured more accurately than the scattered intensity at an arbitrary scattering angle. This prevents from obtaining experimentally the complete scattering diagrams for a single, isolated particle. To the best of our knowledge, there is not any publication discussing this issue in the optical range. One of the goals of the present paper is to fill the gap. To bypass the difficulty related to the small characteristic scale of the scattering problem, we have performed this study with the help of microwave radiation, centimeter-size particles and specially designed dielectric material. Owing to the scaling invariance of the Maxwell equations, the results obtained are in the one-to-one correspondence with the NIR light scattering by nanoparticles made of Si, Ge, etc. with the same radius-to-wavelength ratio^25^.

## Results

**Figure 1:**
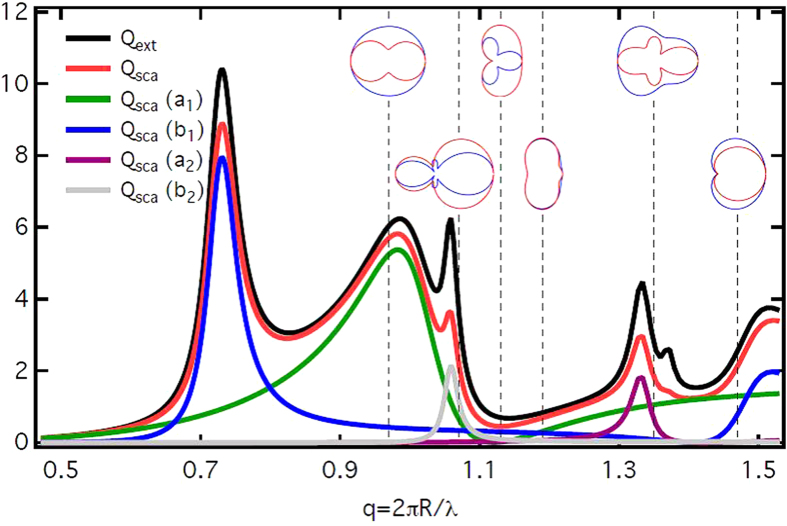
Scattering of a plane, linearly polarized incident electromagnetic wave by a sphere with permittivity *ϵ* = 17.2 + 0.2*i*. Extinction, *Q_ext_* (black line) and scattering, *Q_sca_* (red line ) efficiencies, as functions of the size parameter q = 2πR/λ calculated according to the exact Mie solution^16^. The corresponding contributions of the partial electric dipolar (*Q_sca_*(a_1_) – green line) , magnetic dipolar (*Q_sca_*(b_1_) – blue line), electric quadrupolar (*Q_sca_*(a_2_) – magenta line) and magnetic quadrupolar (*Q_sca_*(b_2_) – grey line) efficiencies to *Q_sca_* are also shown. Vertical dashed lines mark the specific cases which will be discussed in detail in the text. For each of these lines, insets show the scattering diagrams for two perpendicular polarizations of the scattered field: S (blue line) corresponds to the field polarized perpendicularly to the scattering plane and P (red line) to the parallel one. The regions with dominant dipolar and quadrupolar scattering are clearly separated (*q* < 1 and *q* > 1, respectively).

The main feature of HRI dielectric particles is that they have the pronounced, well-separated, both electric and magnetic partial Mie resonances. Examples in the VIS-NIR part of the electromagnetic spectrum can be found in the literature for semiconductor materials like Si^18^,^22^,^26^,^27^,^28^,^29^ and Ge^19^. Similar optical constants (high values of the real part of the refractive index together with low values of its imaginary part) are typical for other semiconductors too^20^. As an example, the extinction 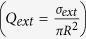 and scattering 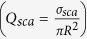 efficiencies, as well as their corresponding partial multipole contributions are presented in Fig. 1 as functions of the size parameter *q* *=* *kR*. Here *σ*_*ext*_ and *σ*_*sca*_ stand for the cross sections, *R* is the radius of the scattering sphere, *k* *=* *2π/λ*, and *λ* denotes the wavelength of the incident radiation in a vacuum (in the case of a particle embedded in a transparent medium the relative refractive index of the particle and the wavenumber in the medium should be employed). Such dependences, *Q*_*ext*_(*q*) and *Q*_*sca*_(*q*), allow to excite the desired partial modes and/or their mixture selectively just by varying *q*. In practice the latter may be achieved either by tuning the frequency of the incident wave *ω* at a fixed *R* or by varying *R* at a fixed *ω.*

Although both *Q*_*ext*_(*q*) and *Q*_*sca*_(*q*) for HRI particles have been analyzed in previous articles for *q* < 1, where the dipole resonances either electric or magnetic dominate (see refs 22, 23, 24, 25, 26, 27, 28, 29)the purpose of Fig. 1 is to illustrate both regimes, *q* < 1 and *q* > 1. For the latter, dipolar and higher order modes coexist and can interfere. Also, we want to show that they have a clear separation, at least for spherical objects. In this research, our objective is the region *q* > 1 where coherent effects between dipolar and higher order modes are pronounced, and *anomalous* scattering effects show up.

**Figure 2:**
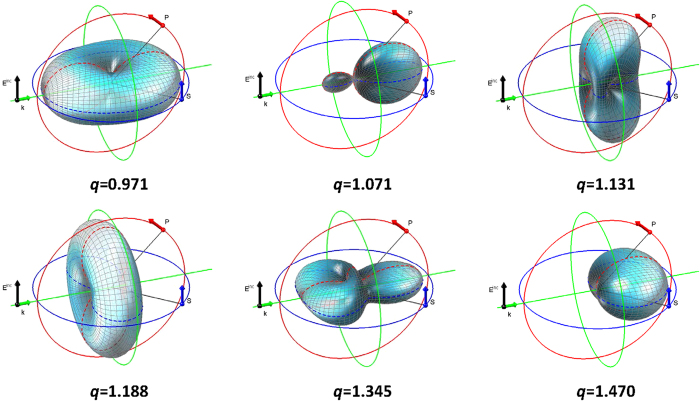
3D calculated scattering diagrams for a sphere with permittivity *ϵ* = 17.2 + 0.2*i*. The incident plane wave is linearly polarized along E^inc^ and its direction of propagation corresponds to k. The linear intensities of the scattered fields at different values of q are calculated according to the exact Mie solution. The scattered field corresponds to the complex summation of the S polarized field and the P polarized field^16^. The S polarized (resp. P polarized) field can be directly obtained when following the blue (resp. red) circle. The values thus collected are indicated with dotted lines. For more details, see Supplementary Information.

Bearing in mind that different partial modes have different angular dependences of the scattered radiation patterns, their selective excitation gives rise to the controlled interference of the scattered electromagnetic waves, which in turn allows tailoring desired scattering diagrams. This opens an almost endless number of possibilities for transfer electromagnetic waves in selected directions, see Fig. 2 as an example.

## Discussion

As the scattering patterns for *q* < 1 are well known, see, *e.g.,*^23^,^24^,^25^,^26^,^27^,^28^,^29^ and refs therein, we will focus on the region *q* ≥ 1. Here the interference between the dipolar and/or quadrupolar modes bring about very useful effects for applications where the control of light guiding is essential^30^,^31^. Let us discuss in detail the scattering patterns at several characteristic values of *q*.

At ***q*** ***=*** **0.971** predominantly the electric dipole mode is excited. The difference between the HRI and the conventional case with refractive index of the order of unity is that at HRI this regime is realized at a much larger value of *R*. Larger elements are easier to fabricate if nanoscale is a concern. The scattering particle in this regime may be utilized as a homogeneous scatterer for the polarization of the scattered radiation parallel to that for the incident wave (the plane containing the blue circle in Fig. 2) and as a semitransparent mirror for the perpendicular one (the plane containing the red circle).

At ***q*** ***=*** **1.071** the scattering pattern is formed by the interference of the electric dipolar and magnetic quadrupolar modes. The interference results in a considerable suppression of the intensity of the scattering in all but forward directions (with only a small scattering in the backward vicinity, see Fig. 2b). From the dipolar point of view it looks similar to the zero-backward Kerker effect^32^ originated in the interference of the two dipolar modes (magnetic and electric) but its nature is different (dipole and quadrupole interference). The spatial concentration of the scattered radiation in this case is also better than that at the Kerker effect. The particle scattering in this regime may act as a subwavelength focusing lens (especially in the “blue” plane).

At ***q*** **=** **1.131** the particle acts as a beam-splitter in the blue plane and as an unusual focussing subwave lens in the “red” plane. Note, that two side-lobes directed almost perpendicular to the wave vector ***k*** of the incident wave in the latter case may provide strong coupling of such particles aligned in an array in a direction perpendicular to the ***k*** plane, which is extremely important for nanoantennas.

At ***q*** **=** **1.188** the particle redirects the incident beam into the plane perpendicular to the vector ***k***.

At ***q*** **=** **1.345** it concentrates the scattered radiation into narrow solid angles.

At ***q*** **=** **1.470** we observe a repetition of the Kerker effect, discussed in a previous publication by some of the authors but at larger values of *q*^25^. Note, that such, almost periodic, repetition of the scattering patterns with an increase in *q* is an intrinsic feature of HRI scatterers. This is very useful to overcome some difficulties in the fabrication of super-diminutive elements for subwavelength optics.

It should be stressed that a selective excitation of desired electromagnetic modes in a scatterer has been discussed before, see, *e.g.,*^12^ and refs therein. However, in these works the excitation is achieved by utilization of either particles with complicated shape and/or structure, or a spatially inhomogeneous source with the characteristic scale of the inhomogeneity of the order of the size of the scatterer, or even smaller than that. Such an experimental approach is always a serious problem at the nanoscale range. In contrast, in our case, the excitation is performed in a simple spatially uniform sphere by a spatially homogeneous plane wave and may be readily realized.

## Methods

**Figure 3:**
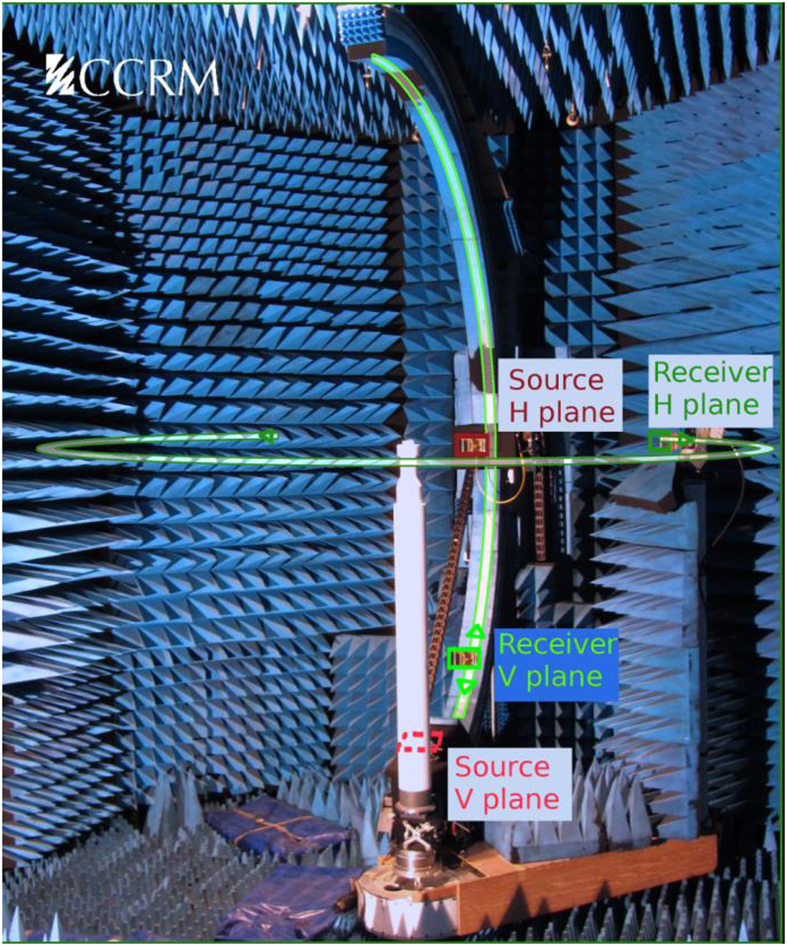
Experimental microwave setup. (**a**) Photograph of the real experimental setup, fully embedded in an anechoic chamber, designed to measure the scattering patterns of a single subwavelength spherical scatterer positioned on the central polystyrene mast. Co-polar scattered fields measurements for the two polarization cases (S and P) are performed either in the Vertical plane (named “V” in the following figures) or the Horizontal plane (named “H”), as schematized by the positions of the various antennas.

To get the experimental evidence of the discussed effects, a series of measurements has been conducted. The experimental setup is shown in Fig. 3. A linearly polarized plane electromagnetic wave of the centimeter-ranged wavelength was scattered by a spherical particle with *R* *=* *9* mm and *ε* = 17.2 + 0.2*i*. Measuring the scattered field in this case is a difficult task since the results may be strongly affected by echoes, stray signals, drift problems, etc. It should be stressed also that the scattered field cannot be measured directly – the field measured by the receiver is a superposition of the incident and scattered ones. To obtain the scattering pattern, the complex subtraction of the fields measured with and without the particle must be performed. It is a complicated experimental problem because the ratio signal/background in this procedure sometimes is less than 1/3000. To overcome these difficulties, an original sophisticated experimental technique has been developed.

The experimental setup allows to measure the scattered fields (both in magnitude and phase thanks to the use of a vector network analyzer with an associated calibration procedure) on a spherical surface of nearly 4 m in diameter, surrounding the scattering target. The scattering sphere is positioned at the center of the measurement sphere, on a rotating polystyrene mast almost transparent in the frequency range of study. For more details see^25^,^33^,^34^ and Supplementary Information.

**Figure 4:**
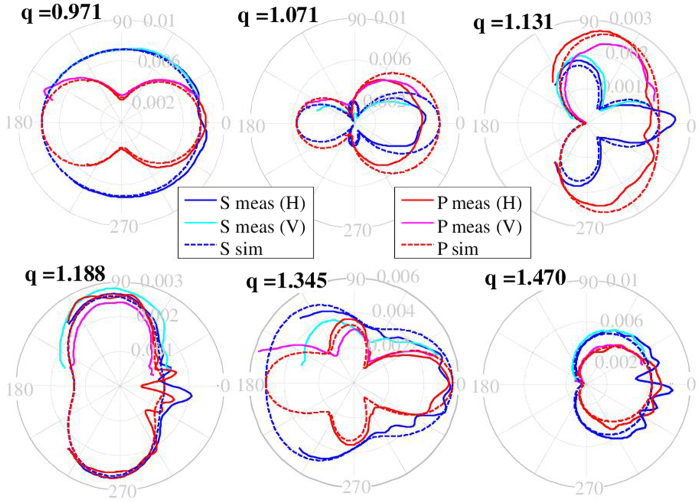
Linear polar representation of the amplitude of the electric field scattered by a spherical scatterer of radius 9 mm and permittivity *ϵ* = 17.2 + 0.2*i* at different values of the size parameter *q* (indicated above the patterns). Simulated results are represented with red or blue dotted lines (labeled as “sim” in the legend). Cyan and blue lines correspond to S polarization (perpendicular to the scattering (drawing) plane), red and magenta lines to P polarization (parallel to the scattering (drawing) plane). Experimental results are plotted with continuous lines and labeled as “meas”. Measurements made in the horizontal and vertical configurations respectively, are labeled “V” and “H” in the legend respectively. See also Figs 2 and 3.

The measured scattering diagrams, corresponding to those presented in Fig. 2, for the two independent planes and their comparison with the ones calculated based upon the exact Mie solution are shown in Fig. 4. The excellent agreement of the theory and experiment is clearly seen.

The obtained theoretical and experimental results provide solid grounds to conclude that relatively cheaply and easily fabricated spatially homogeneous particles with HRI may play a role of new universal multifunctional elements in various optical devices, such as optical circuits, nanoantennas, etc. The new important features of these elements are (i) their ability to operate by being excited by a simple plane electromagnetic wave and (ii) the opportunity to switch between the different functions of these elements just by relatively small (within 30–40%) changes in the frequency of the incident wave (at a fixed size of the particles), by the corresponding changes in their sizes (at a fixed frequency) or by playing with the polarization of the incident wave (see Supplementary Information).

## Additional Information

**How to cite this article**: Tribelsky, M. I. *et al.* Small Dielectric Spheres with High Refractive Index as New Multifunctional Elements for Optical Devices. *Sci. Rep.* **5**, 12288; doi: 10.1038/srep12288 (2015).

## Supplementary Material

Supplementary Information

Supplementary Video 1
