# Supplementary material for: Small Dielectric Spheres with High Refractive Index as New Multifunctional Elements for Optical Devices
Source: Sci Rep. 2015 Jul 23;5:12288. doi: 10.1038/srep12288 (PMC5378880; doi:10.1038/srep12288)
Supplement: Supplementary Information [file srep12288-s1.pdf]

Supplementary Information

Small Dielectric Spheres with High Refractive Index as New Multifunctional Elements for Optical Devices

Michael I. Tribelsky[1,2)], Jean-Michel Geffrin[3)], Amelie Litman[3)], Christelle Eyraud[3)]
Fernando Moreno[4,*)]

1) Lomonosov Moscow State University, Russia
2) Technical University MIREA, Moscow, Russia
3) Aix-Marseille Université, CNRS, Centrale Marseille, Institut Fresnel UMR 7249, 13013 Marseille, France
4) Group of Optics. Department of Applied Physics. University of Cantabria, Spain

**Correspondence to:* morenof@unican.es

The Scattered Field

When a linearly-polarized plane wave $\boldsymbol{E}^{inc}$, defined by its polarization direction and its wave-vector $\boldsymbol{k}$, illuminates a homogeneous non-magnetic (μ=1) sphere, a scattered field $\boldsymbol{E}^{sca}$ is generated whose characteristics depend on the sphere dimension q and on its permittivity ε. The total field is given by $\boldsymbol{E}^{tot} = \boldsymbol{E}^{inc} + \boldsymbol{E}^{sca}$ (from an experimental point of view, $\boldsymbol{E}^{sca}$ is not measured directly but results from the complex subtraction of the two measured fields, $\boldsymbol{E}^{tot}$ and $\boldsymbol{E}^{inc}$). For the sake of simplicity, the sphere is located at the center of the coordinate system, the wave-vector is such that $\boldsymbol{k} = k\boldsymbol{e}_z$ and $\boldsymbol{E}^{inc} = E_x^{inc}\boldsymbol{e}_x$. Due to the topology of the scatterer, a spherical coordinate system with unitary vectors $\boldsymbol{e}_r(r,\theta,\varphi)$, $\boldsymbol{e}_\theta(r,\theta,\varphi)$ and $\boldsymbol{e}_\varphi(r,\theta,\varphi)$ is more appropriate [1]. The incident field is now written as

$$\boldsymbol{E}^{inc} = E_\theta^{inc}\boldsymbol{e}_\theta(r,0,\varphi) - E_\varphi^{inc}\boldsymbol{e}_\varphi(r,0,\varphi),$$

with $E_\theta^{inc} = E_x^{inc}cos(\varphi)$ and $E_\varphi^{inc} = E_x^{inc}sin(\varphi)$. As the sphere is assumed to be non-depolarizing, its associated scattered field is

$$\boldsymbol{E}^{sca} = E_P^{sca}\boldsymbol{e}_\theta(r,0,\varphi) - E_S^{sca}\boldsymbol{e}_\varphi(r,0,\varphi),$$

where the P and S components of the field are computed respectively, thanks to the amplitude scattering matrix $\boldsymbol{S}$ which depends on q, ε and the spherical coordinates [1],

$$E_P^{sca} = \frac{exp(ikr)}{-ikr}S_2E_\theta^{inc},$$

and

$$E_S^{sca} = \frac{exp(ikr)}{-ikr} S_1 E_\varphi^{inc}.$$

Figure 2 shows the behavior of $|\boldsymbol{E}^{sca}|$ for various values of q. In Figure 4, the amplitude of $|E_P^{sca}|$ and $|E_S^{sca}|$ are provided. They are either computed or measured. In practice, they are not measured directly but obtained from the complex subtraction of the total field, $\boldsymbol{E}^{tot} = \boldsymbol{E}^{inc} + \boldsymbol{E}^{sca}$, measured in presence of the target and the incident field $\boldsymbol{E}^{inc}$. Owing to the orthogonality properties of the unitary vectors $\boldsymbol{e}_\theta$ and $\boldsymbol{e}_\varphi$, each part of the field component can be measured independently with a linearly-polarized receiving antenna. The S component is obtained when the receiver is polarized along $\boldsymbol{e}_x$ and moves along the blue circle (ϕ = π/2). The P component is measured when the receiver is polarized along $\boldsymbol{e}_\theta$ and moves along the red circle (ϕ= 0). If the emitting antenna is shifted, the entire scene is shifted as well in a similar manner. This rotation property enables to acquire the fields either in the V plane or the H plane as depicted in Figure 3.

The experimental setup and measurements

The experimental setup shown in Fig. 3 allows measuring scattered fields (both in magnitude and phase thanks to the use of a vector network analyzer with an associated calibration procedure) on a spherical surface of nearly 4 m in diameter, surrounding the scattering target under test. This target is positioned at the center of the measurement sphere, on a rotating polystyrene mast almost transparent in the frequency range of study.

By pivoting the linearly polarized broadband ridged horn antennas (ARA DRG118), s-polarized (resp. p-polarized) waves perpendicular (resp. parallel) to the scattering plane and named S and P respectively in the following, can be emitted and received (see Fig. 3). In order to compensate for some otherwise experimentally inaccessibly angular ranges (for example the backward scattered field which is not reachable in the horizontal arrangement), the emitting and receiving antennas have been placed on the different positioning devices shown in Fig. 3 to measure the scattering patterns either in the vertical plane (*V*) or in the horizontal plane (*H*). Furthermore, thanks to some overlap in the resulting angular ranges, it provides crosscheck comparisons which fully assess the validity of the overall measurement protocol.

The measurement of the scattered fields is long and arduous, especially when the target presents dimensions which are small with respect to the wavelength of the incoming wave. This field is not directly measurable and can only be deduced from the subtraction of the complex values of the fields acquired with and without the target. Any perturbation from parasitic echoes, stray signals, and drift problems will affect the results. These problems are exacerbated at higher frequencies and require specific calibration and post-processing procedures. These steps have been

previously detailed in [2, 3, 4].

In the present work, the scattering pattern of a spherical dielectric particle of radius R = 9 mm, already used in [2] has been measured with the same frequency range [3.5-8.5] GHz. (i. e. q between 0.6 and 1.6). This sphere is made of ECCOSTOCK-HIK (from Emerson and Cuming. http://www.eccosorb.com/)) and its permittivity is similar to that of some semiconductor materials (Si, Ge) in the near IR, with almost no dispersion in the frequency range of interest and very low losses. The permittivity estimation of this material has been further refined by minimizing (thanks to a non-linear optimization scheme) the discrepancy between the Mie calculations and the measured scattered field of a bigger sphere made from the same material. The permittivity was estimated to be $\varepsilon = 17.2 + 0.2i$ and this value was employed in all the computations and analysis presented in this paper.

Additional improvements were performed on the measurement process as, for example, the employed time gating filters were more restricted. The major improvement was the replacement of the vector network analyzer (VNA) with a more recent one (ZNB from Rohde & Schwarz) with two external synthesizers. A fine tuning of the VNA parameters has allowed to remove most of the saturation effect in the forward scattering region without any visible increase of the noise in the regions of low-level fields. Thus, various measured scattered fields patterns can be presented.

References

[1] Bohren, G. F. & Huffman, D. R. *Absorption and scattering of light by small Particles* (Wiley and Sons, 1983).

[2] Geffrin, J. M., et al., Magnetic and electric coherence in forward- and back-scattered electromagnetic waves by a single dielectric subwavelength sphere, *Nat. Commun.* **3**, 1171, doi:10.1038/ncomms2167 (2012).

[3] Eyraud, C., Geffrin, J.-M., Litman, A., Sabouroux, P. & Giovannini, H. Drift correction for scattering measurements, *Appl. Phys. Lett.*, **89**, 244104, (2006)

[4] Geffrin, J.-M., Eyraud, C., Litman, A. & Sabouroux, P. Optimization of a Bistatic Microwave Scattering Measurement Setup: From High to Low Scattering Targets, *Radio Science*, **44**, RS00383, (2009)
